# Triglyceride-Glucose Index for Early Prediction of Nonalcoholic Fatty Liver Disease: A Meta-Analysis of 121,975 Individuals

**DOI:** 10.3390/jcm11092666

**Published:** 2022-05-09

**Authors:** Azizullah Beran, Hazem Ayesh, Mohammed Mhanna, Waseem Wahood, Sami Ghazaleh, Ziad Abuhelwa, Wasef Sayeh, Nameer Aladamat, Rami Musallam, Reem Matar, Saif-Eddin Malhas, Ragheb Assaly

**Affiliations:** 1 Department of Internal Medicine, University of Toledo, Toledo, OH 43606, USA; hazem.ayesh@utoledo.edu (H.A.); mohammed.mhanna@utoledo.edu (M.M.); sami.ghazaleh@utoledo.edu (S.G.); ziad.abuhelwa@utoledo.edu (Z.A.); wasef.sayeh@utoledo.edu (W.S.); saif-eddin.malhas@utoledo.edu (S.-E.M.); ragheb.assaly@utoledo.edu (R.A.); 2 Dr. Kiran C. Patel College of Allopathic Medicine, Nova Southeastern University, Davie, FL 33314, USA; ww412@mynsu.nova.edu; 3 Department of Neurology, University of Toledo, Toledo, OH 43606, USA; nameer.aladamat@utoledo.edu; 4 Department of Internal Medicine, St. Vincent Charity Medical Center, Cleveland, OH 44115, USA; msallam93@gmail.com; 5 Department of Gastroenterology and Hepatology, Mayo Clinic, Rochester, MN 55905, USA; reem-matar@hotmail.com

**Keywords:** triglyceride-glucose index, nonalcoholic fatty liver disease, NAFLD, NASH, hepatic steatosis

## Abstract

Insulin resistance (IR) is a major contributor to the pathogenesis of nonalcoholic fatty liver disease (NAFLD). The triglyceride-glucose (TyG) index has recently gained popularity for the assessment of IR and NAFLD due to its ease of acquisition and calculation. Therefore, we conducted this systematic review and meta-analysis to summarize the existing studies in the literature and provide a quantitative assessment of the significance of the TyG index in predicting the incidence of NAFLD. A comprehensive literature search in PubMed, EMBASE, and Web of Science databases from inception until 25 March 2022 was conducted. Published observational studies that evaluated the association between TyG index and NAFLD among the adult population and reported the hazard ratio (HR) or odds ratio (OR) for this association after multivariate analysis were included. The random-effects model was used as the primary statistical analysis model in the estimation of pooled ORs and HRs with the corresponding confidence intervals (CIs). A total of 17 observational studies, including 121,975 participants, were included. For studies analyzing the TyG index as a categorical variable, both pooled OR (6.00, CI 4.12–8.74) and HR (1.70, CI 1.28–2.27) were significant for the association between TyG index and incident NAFLD. For studies analyzing the TyG index as a continuous variable, pooled OR (2.25, CI 1.66–3.04) showed similar results. Consistent results were obtained in subgroup analyses according to the study design, sample size, ethnicity, and diabetic status. In conclusion, our meta-analysis demonstrates that a higher TyG index is associated with higher odds of NAFLD. TyG index may serve as an independent predictive tool to screen patients at high risk of NAFLD in clinical practice, especially in primary care settings. Patients with a high TyG index should be referred for a liver ultrasound and start intense lifestyle modifications. However, further large-scale prospective cohort studies are necessary to validate our findings.

## 1. Introduction

Nonalcoholic fatty liver disease (NAFLD) is the most prevalent liver disease in Western countries. The global prevalence of NAFLD is estimated to be approximately 25%, which is projected to increase to 33.5% by 2030 [1]. NAFLD can complicate into nonalcoholic steatohepatitis, cirrhosis, or even hepatocellular carcinoma [1]. NAFLD is also considered a risk factor for extrahepatic diseases such as cardiovascular disease (CVD), chronic kidney disease (CKD), colorectal cancer, type 2 diabetes mellitus (T2DM), and osteoporosis [1]. There is no approved pharmacological treatment available for NAFLD currently except for lifestyle changes [2]. Thus, early detection of patients at risk for NAFLD using simple and effective diagnostic methods is crucial.

The exact pathogenesis of NAFLD is not fully elucidated. However, insulin resistance (IR) has been hypothesized to play a key role in the development of NAFLD [3]. IR is characterized by impaired glucose uptake and oxidation [3]. The triglyceride-glucose (TyG) index, derived by the formula ln (fasting triglycerides (mg/dL) fasting plasma glucose (mg/dL)/2), has recently gained popularity among researchers due to its greater performance in estimating IR compared with a homeostatic model assessment of IR (HOMA-IR) [4,5,6]. Furthermore, due to its ease of acquisition and calculation, it has been widely accepted and used in clinical practice to assess IR [7,8].

In 2016, Simental-Mendia et al. [9] showed that the TyG index was the best test for screening simple hepatic steatosis and nonalcoholic steatohepatitis. Since then, accumulating cross-sectional and cohort studies evaluating the relationship between the TyG index and the incidence of NAFLD have been published [10,11,12,13,14]. However, the results of previous studies on the association between the TyG index and incident NAFLD are inconsistent [10,14,15]. Therefore, we conducted this systematic review and meta-analysis to summarize the existing studies in the literature and provide a quantitative assessment of the significance of the TyG index in predicting the incidence of NAFLD.

## 2. Materials and Methods

We conducted this systematic review and meta-analysis based on the guidelines of the Preferred Reporting Items for Systematic Reviews and Meta-analysis [16] and Meta-analysis of Observational Studies in Epidemiology [17].

### 2.1. Data Sources and Search Strategy

We performed a systematic search for published studies indexed in PubMed, EMBASE, and Web of Science databases from inception to 25 March 2022. We also performed a manual search for additional relevant studies using references from the included articles. The following search terms were used: (“triglyceride-glucose index”) and (“nafld”, “nash”, or “nonalcoholic fatty liver disease”). Supplementary Table S1 describes the full search term used in each database searched. Two investigators (A.B. and H.A.) independently performed the literature search, screened using a priori criteria, and shortlisted the studies for final review. The bibliographic software EndNote was used for screening. Any discrepancies were resolved by a third reviewer (M.M.).

### 2.2. Eligibility Criteria

The inclusion criteria for the studies were (1) observational studies (cohort, case–control, or cross-sectional) published as full-length articles in the English language; (2) included adult population; (3) evaluated the association between the TyG index and NAFLD; and (4) reported the hazard ratio (HR) or odds ratio (OR) for this association after multivariate analysis and adjustment of potential confounding factors. Studies were included regardless of how NAFLD development was measured. We excluded studies when TyG index data could not be extracted or were not reported, studies on children (age < 18 years old), studies that reported data based on univariate analysis rather than multivariate analysis, or animal studies. We also excluded unpublished data, including preprints and conference abstracts.

### 2.3. Data Extraction

The following data were extracted from the studies: first author name, publication year, country of origin, study design, participants’ characteristics, exclusion criteria, total participants’ number, gender and age of patients, body mass index (BMI), TyG index analysis (categorized or continuous), diagnosis of NAFLD, confounding variables adjusted in the multivariate analyses, and follow-up duration. Two investigators (A.B. and H.A.) independently extracted the data from the included studies. Microsoft Excel 2019 (Microsoft Corporation, Redmond, WA, USA) was used for data extraction. Any discrepancies were resolved by consensus.

### 2.4. Outcomes of Interest

Our primary outcome of interest was the association between the TyG index and the incidence of NAFLD.

### 2.5. Statistical Analysis

The association between the TyG index and the incident NAFLD was assessed as either categorized or continuous variables such as adjusted OR or adjusted HR. If the TyG index was assessed as a categorical variable, the OR or HR comparing the highest group of TyG index to the lowest group of TyG index was extracted. If the TyG index was assessed as a continuous variable, the OR or HR of the NAFLD incidence per 1-unit increment of the TyG index was extracted. If more than one model was used for the multivariate analysis, the one with the most fully adjusted parameters was selected. Due to the high incidence of NAFLD in our study, HRs and ORs were pooled separately. The random-effects model was used as the primary statistical analysis model in the estimation of pooled ORs and HRs with the corresponding confidence intervals (CI). Heterogeneity was assessed using the Higgins I^2^ index, where I^2^ values > 50% implied the presence of substantial heterogeneity [18]. Statistical analysis was performed using Review Manager 5.3 (Cochrane Collaboration, Copenhagen, Denmark, The Nordic Cochrane Centre) and Comprehensive Meta-Analysis (Biostat, Englewood, NJ, USA).

A leave-one-out sensitivity analysis was performed to test the robustness of the results for outcomes reported by >5 studies. Finally, we performed subgroup analyses to assess the impact of study and participant characteristics (study design, country of population, sample size, and diabetic status) on the outcome of interest.

### 2.6. Quality Assessment

The Newcastle Ottawa Quality Assessment Scale (NOS) was used to assess the methodology of the observational studies based on the selection of the study groups, comparability of study groups, and ascertainment of exposure/outcome [19]. Studies with total scores of ≥6 were considered to have a low risk of bias. We were not able to conduct an assessment of publication bias due to the small number of studies (<10 for all effect estimates).

We used the Grading of Recommendations Assessment, Development and Evaluation (GRADE) methodology to assess the quality of evidence [20]. The GRADE approach classifies the quality of evidence as high, moderate, low, or very low. The quality of evidence for randomized controlled trials begins with high confidence, while it begins with low confidence for observational studies. The methodologic quality (risk of bias), directness of evidence, heterogeneity, the precision of effect estimates, and publication bias are also appraised. We utilized GRADEpro (https://www.gradepro.org/, accessed on 2 April 2022) to grade evidence quality.

## 3. Results

### 3.1. Study Selection

**Figure 1:**
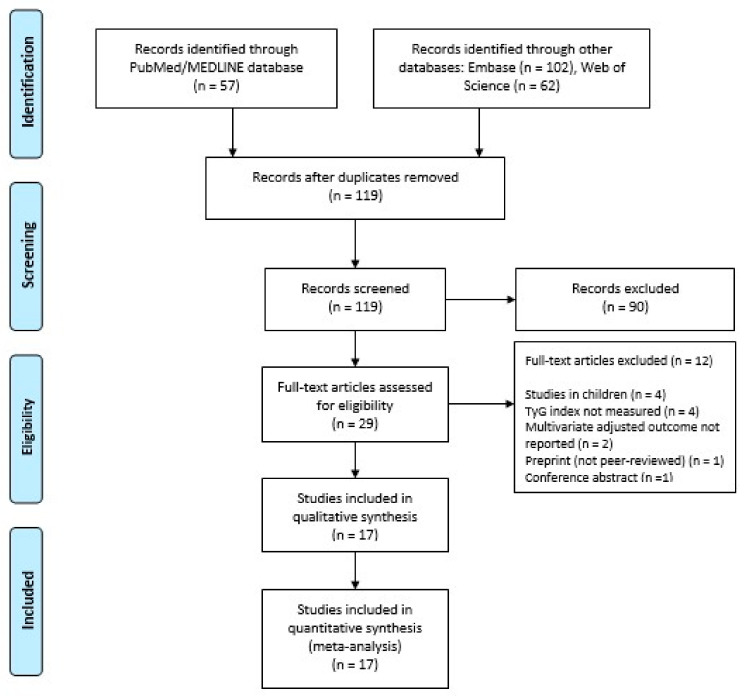
PRISMA flow diagram for the selection of studies.

Our search strategy retrieved a total of 221 studies. Among these, 29 studies were eligible for systematic review. Subsequently, we excluded 12 studies because of lack of measurement of TyG index, lack of reporting multivariate-adjusted outcome, the inclusion of children, lack of peer-review (preprints), or conference abstracts. Eventually, 17 studies [10,11,12,13,14,15,21,22,23,24,25,26,27,28,29,30,31] met our inclusion criteria and were included in the meta-analysis. Figure 1 shows the PRISMA flowchart that illustrates how the final studies were selected.

### 3.2. Study and Participants’ Characteristics

Table 1 shows the study and participants’ characteristics of the studies included in the meta-analysis. A total of 17 studies [10,11,12,13,14,15,21,22,23,24,25,26,27,28,29,30,31] were included in the meta-analysis. All the included studies were published between January 2017 and March 2022. Based on country of origin, seven studies [10,13,14,28,29,30,31] originated from China, three studies [22,23,26] from Korea, three studies [11,12,15] from Iran, one study [25] from France, one study [24] from Japan, one study [27] from Taiwan, and one study [21] from Turkey. Regarding the design of studies, eleven [10,12,14,15,21,22,26,27,28,29,31] were cross-sectional studies and six [11,13,23,24,25,30] were cohort studies. Of the 17 studies, 13 studies [10,11,12,13,14,21,23,24,26,27,29,30,31] included participants of the general population, while 1 study [22] included patients with chronic kidney disease, 1 study [25] included patients with obesity, 1 study [15] included patients with T2DM, and 1 study [28] included women of the general population.

A total of 121,975 participants were included in our analysis, with a mean age of 50.3 years. The proportion of males ranged from 23.5% to 83.3%. The mean BMI ranged from 21.2 to 42. The incidence of NAFLD in our analysis was 24.9%. Of the 17 studies, NAFLD was diagnosed by ultrasound in 14 studies [10,12,13,14,21,22,23,24,26,27,28,29,30,31] while 1 study [15] used elastography, 1 study [25] used liver biopsy, and 1 study [11] used fatty liver index to diagnose NAFLD. The baseline TyG index was analyzed as a categorical variable in 12 studies [10,11,13,14,15,21,23,24,26,28,29,30] and as a continuous variable in 5 studies [12,22,25,27,31]

### 3.3. TyG Index and Incidence of NAFLD

#### 3.3.1. TyG Index Analyzed as a Categorical Variable

In total, 12 studies [10,11,13,14,15,21,23,24,26,28,29,30], which included 104,514 participants, analyzed the TyG index as a categorical variable.

**Figure 2:**
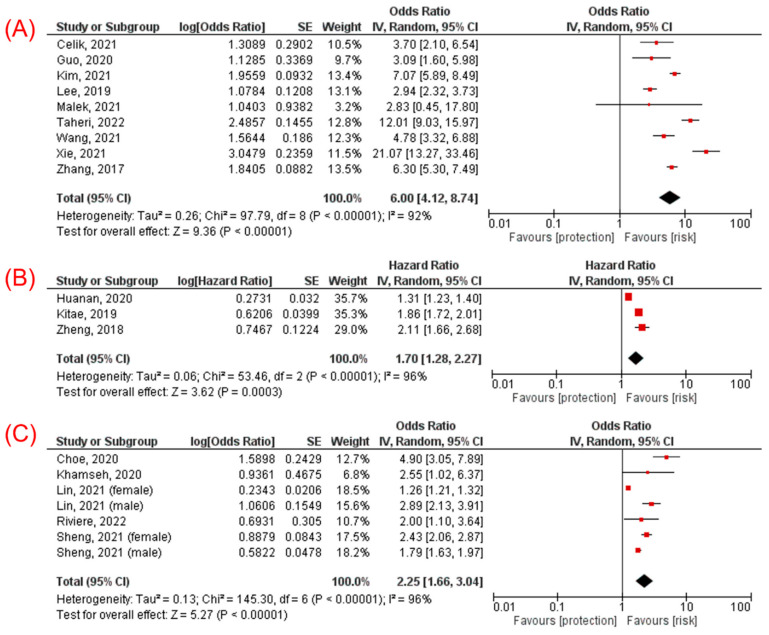
(**A**) Forest plot evaluating association between TyG index (analyzed as a categorical variable and presented as OR) and NAFLD [10,11,14,15,21,23,26,28,29]; (**B**) forest plot evaluating association between TyG index (analyzed as a categorical variable and presented as HR) and NAFLD [13,24,30]; (**C**) forest plot evaluating association between TyG index (analyzed as a continuous variable and presented as OR) and NAFLD [12,22,25,27,31].

Of the 12 studies, 9 studies [10,11,14,15,21,23,26,28,29], which included 39,196 participants, presented data as adjusted OR. The pooled results of nine studies showed participants with the highest TyG index category had a significantly increased odds of having NAFLD, compared with participants with the lowest TyG index category (OR 6.00, 95% CI 4.12–8.74, *p* < 0.00001, I^2^ = 92%, Figure 2A).

**Figure 3:**
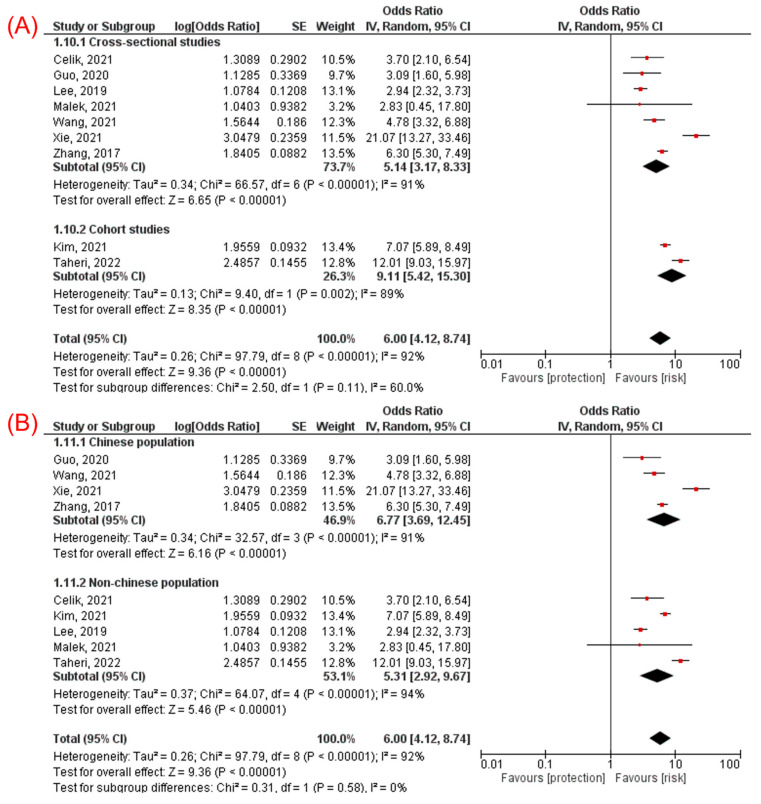
Subgroup analyses for the association between TyG index and NAFLD: (**A**) subgroup analysis according to the study design [10,11,14,15,21,23,26,28,29] and (**B**) subgroup analysis according to the ethnicity of the population [10,15,21,23,26].

**Figure 4:**
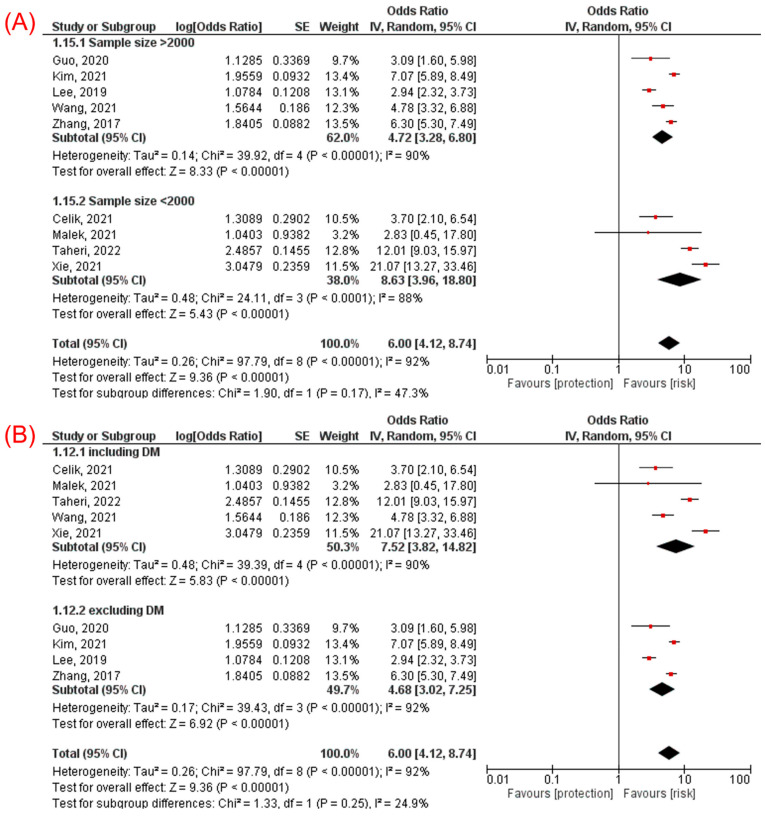
Subgroup analyses for the association between TyG index and NAFLD: (**A**) subgroup analysis according to the sample size [10,11,14,15,21,23,26,28,29] and (**B**) subgroup analysis according to the diabetic status of the population [10,11,14,15,21,23,26,28,29].

Subgroup analyses showed consistent association in cross-sectional (OR 5.14, 95% CI 3.17–8.33, *p* < 0.00001) and cohort (OR 9.11, 95% CI 5.42–15.30, *p* < 0.00001, Figure 3A) studies, in Chinese (OR 6.77, 95% CI 3.69–12.45, *p* < 0.00001) and non-Chinese (OR 5.31, 95% CI 2.92–9.67, *p* < 0.00001, Figure 3B) population, in studies with sample size ≥ 2000 participants (OR 4.72, 95% CI 3.28–6.80, *p* < 0.00001) and sample size < 2000 (OR 8.63, 95% CI 3.96–18.80, *p* < 0.00001, Figure 4A), and in studies including participants with diabetes mellitus (OR 7.52, 95% CI 3.82–14.82, *p* < 0.00001) and excluding participants with diabetes mellitus (OR 4.68, 95% CI 3.02–7.25, *p* < 0.00001, Figure 4B).

Of the 12 studies, 3 studies [13,24,30], which included 65,218 participants, reported data as adjusted HR. The pooled results of these studies were consistent (HR 1.70, 95% CI 1.28–2.27, *p* = 0.0003, I^2^ = 96%, Figure 2B).

#### 3.3.2. TyG Index Analyzed as a Continuous Variable

Five studies [12,22,25,27,31], which included 17,461 participants, analyzed the TyG index as a continuous variable and presented data as adjusted OR. The pooled results were consistent with the TyG index analyzed as a continuous variable (OR 2.25, 95% CI 1.66–3.04, *p* < 0.00001, I^2^ = 96%, Figure 2C).

### 3.4. Sensitivity Analysis

A leave-one-out sensitivity analysis showed similar results (ORs for the TyG index analyzed as a categorical variable), as shown in Supplementary Figure S1.

### 3.5. Quality Assessment

Methodological assessment scores of the included studies based on NOS are summarized in Supplementary Table S2. There was a low risk of bias for all 17 studies (Supplementary Table S2). According to the GRADE approach, the level of quality of evidence was very low for the outcome of interest (association between the TyG index and incidence of NAFLD) because of the study design (all the included studies were observational studies) and substantial heterogeneity across the included studies.

## 4. Discussion

In this meta-analysis of observational studies, we systematically assessed the association between the TyG index and the incidence of NAFLD. We found that, compared with individuals with the lowest TyG index category, those with the highest category were independently associated with an increased incidence of NAFLD. Consistent results were obtained in subgroup analyses according to the study design, sample size, ethnicity, and diabetic status. Furthermore, a meta-analysis with the TyG index analyzed as a continuous variable also demonstrated that a higher TyG index at baseline was independently associated with an increased risk of the subsequent incidence of NAFLD. Based on our study results, a higher TyG index may be used as an independent predictor of an increased risk of NAFLD incidence. The TyG index may serve as a simple and useful indicator for risk assessment of NAFLD in clinical practice.

Our results were consistent with a large prospective cohort study by Kitae et al. [24], which showed that the TyG index is significantly associated with incident NAFLD based on ultrasound in the general adult population (HR 1.86, 95% CI 1.72–2.01). Similar to our findings, a cohort study by Riviere et al. [25], which used a robust method for detecting NAFLD by liver biopsy among patients with obesity, showed consistent results of a strong association between the TyG index and NAFLD (OR 2.00, 95% CI 1.72–2.01).

NAFLD is becoming a worldwide silent epidemic that has significant health and economic burden [32]. NAFLD is largely ignored by patients since there are no evident clinical signs. However, if left untreated, NAFLD can lead to serious complications such as nonalcoholic steatohepatitis (NASH), cirrhosis, and even hepatocellular carcinoma [33]. Therefore, early detection and intervention for NAFLD patients are crucial to prevent the subsequent complications of NAFLD and other associated chronic diseases such as DM.

IR is the major contributor to the pathogenesis of NAFLD via increased delivery of free fatty acids to the liver, inadequate fatty acid oxidation, and increased de novo lipogenesis [34]. Furthermore, the TyG index outperforms even HOMA-IR in predicting NAFLD. The hyperinsulinemic–euglycemic clamp test is the gold standard for IR testing; however, it is time-consuming and costly [35]. Another approach for IR testing is HOMA-IR, which needs to measure serum insulin levels; however, large-scale screening in primary care settings using HOMA-IR is not feasible [35]. TyG index is a new convenient surrogate marker for IR that has gained ground recently due to its simplicity in calculating [36]. In addition, the TyG index showed superiority over HOMA-IR in predicting IR [5]. Furthermore, the TyG index was found to be the best test to screen for simple steatosis and NASH, compared with other indices for NAFLD such as SteatoTest, NashTest, and fatty liver index [9]. In addition, these indices are not widely used in clinical practice, owing to their complexity and difficulty in calculations, as well as their high cost [9]. TyG index is unique in being easy to calculate and thus can be a practical tool in primary care settings to screen for NAFLD. Furthermore, the TyG index outperforms even HOMA-IR in predicting NAFLD [26]. According to Zhang et al. [10], the TyG threshold of 8.5 was highly sensitive for detecting NAFLD patients and concluded that this threshold might be adequate to diagnose NAFLD in the general adult population. Furthermore, previous studies have demonstrated a strong dose–response association between the TyG index and NAFLD when categorizing the TyG index into quartiles [13,14,23,26]. Guo et al. [14] showed that the prevalence of NAFLD increased from 30.9% to 53.3%, to 71.7%, to 86.4% across increasing TyG quartiles (Q1, Q2, Q3, and Q4, respectively; *p*-value for trend < 0.001). Huanan et al. [13] further demonstrated that the higher the level of the TyG index, the greater the incidence of NAFLD, regardless of whether the TyG index was analyzed as a continuous or categorical variable.

TyG index may be associated with the severity of hepatic steatosis and liver fibrosis. However, our analysis could not assess the association between the TyG index and liver fibrosis due to the limited number of studies reporting the multivariate-adjusted ORs of this association [12,14,25,37]. Khamseh et al. [12] and Riviere et al. [25] analyzed the TyG index as a continuous variable, while Guo et al. [14] and Tutunchi et al. [37] analyzed the TyG index as a categorical variable. A study by Guo et al. revealed that, with each TyG index quartile, there was a significant rise in the percentage of liver fibrosis (based on liver stiffness measurement) among patients with NAFLD (Q1: 13.5%, Q2: 17.6%, Q3: 18.8%, and Q4: 26.1%; *p* < 0.001) [14]. When comparing the second, third, and fourth quantiles of the TyG index to the first quantile of the TyG index, the multivariate-adjusted ORs (95% CI) were 1.98 (1.33–2.22), 2.33 (2.09–2.94), and 3.44 (2.63–4.25), respectively, based on the NAFLD fibrosis score [37]. Another cross-sectional study showed that the TyG index was significantly associated with the severity of liver fibrosis proven by liver biopsy. The mean TyG index value for mild fibrosis was 8.95 ± 0.08, 9.32 ± 0.24 for moderate fibrosis and 9.35 ± 0.85 for severe fibrosis (*p* < 0.0001) [38]. Riviere et al. [25] reported that the TyG index could also predict the presence of NASH (multivariate-adjusted OR was 4.7, 95% CI 2.3–9.5). Further studies are needed to assess the association between the TyG index and NAFLD progression to NASH and liver fibrosis.

Several limitations of this study should be acknowledged. First, all the included studies were observational in nature, and most of them were cross-sectional and retrospective in nature with their inherent selection and confounding biases. Therefore, more large-scale prospective cohort studies are needed to confirm our results. Second, although we included studies with multivariate analysis only, we cannot rule out unadjusted residual factors that may confound the TyG index and NAFLD association, such as dietary factors. Third, even though the random-effects model was used in our analysis, substantial heterogeneity was noted across the studies. We were unable to determine the source of substantial heterogeneity between the studies despite performing sensitivity and subgroup analyses. However, this significant heterogeneity might be driven by differences in participants’ comorbidities, concurrent medications, dietary factors, and cut-off values for the TyG index. Fourth, most studies used ultrasound to diagnose NAFLD, which may be less accurate, compared with liver biopsy. Ultrasound, on the other hand, has shown high sensitivity and specificity in the diagnosis of NAFLD [39]. Lastly, Finally, because this study is a meta-analysis of observational studies, a causal relationship between a higher TyG index and NAFLD cannot be inferred.

Despite the limitations, our study has several strengths. First, to our knowledge, this is the first systematic review and meta-analysis to evaluate the association between the TyG index and the incidence of NAFLD. We included 17 observational studies, with a total of 121,975 participants, in this meta-analysis. Second, we performed meta-analyses separately with the TyG index analyzed as a categorical variable, as well as a continuous variable, and consistent results were obtained, indicating that our results were robust. Lastly, our results remained consistent in sensitivity and subgroup analyses, indicating that our results were not driven by a single study or affected by study or participants’ characteristics such as study design, sample size, ethnicity, or diabetic status.

## 5. Conclusions

Our meta-analysis of observational studies demonstrates that a higher TyG index is associated with higher odds of NAFLD. TyG index may serve as an independent predictive tool to screen patients at high risk of NAFLD in clinical practice, especially in primary care settings. Patients with a high TyG index should be referred for a liver ultrasound and start intense lifestyle modifications. However, further large-scale prospective cohort studies are necessary to validate our findings.

## Figures and Tables

**Table 1:** Study and participants’ characteristics of the included studies.

Study, Year	Study Design	Country	Participant Characteristics	Exclusion Criteria	Total Participants, *n*	Male, *n* (%)	Mean Age, Years	Mean BMI, kg/m^2^	Incidence of NAFLD, *n* (%)	Liver Fibrosis, *n* (%)	Diagnosis of NAFLD	TyG Index Analysis	Variables Adjusted	Follow-Up Period
Celik, 2021 [21]	Retrospective CS	Turkey	General population	alcohol abuse, chronic liver disease, acute abdomen, ESRD, sepsis, cancer, and neuropsychiatric diseases	986	232 (23.5)	55.9	NR	470 (47.7)	NR	Ultrasound	Categorized (Step 5a:Step 1a)	Age, ALT, AST, LDL, HDL, DM, and prediabetes	NR
Choe, 2020 [22]	CS	Korea	Patients with CKD	alcohol abuse, viral hepatitis, other chronic liver diseases	819	682 (83.3)	64.6	25	140 (17.1)	NR	Ultrasound	Continuous	Age, gender, BMI, HTN, DM, HLD, eGFR, log creatinine, RRT, EPO use, log WBC, Hb, PLT, log AST, log ALT, log TB, albumin, log CRP, log TG, TC, HbA1C, log FG	NR
Guo, 2020 [14]	CS	China	General population	alcohol abuse, HLD, DM, viral hepatitis, chronic liver disease, hyperthyroidism, kidney disease	4784	3231 (67.5)	48.96	24.9	2902 (60.7)	550 out of 2352 (23.4%)	Ultrasound	Categorized (Q4–Q1)	BMI, HbA1c ALT, AST and GGT	NR
Huanan, 2020 [13]	RC	China	General population	alcohol abuse, viral hepatitis, chronic liver disease, including NAFLD	46,693	22,932 (49.1)	68.1	24.82	5660 (12.1)	NR	Ultrasound	Categorized (Q4–Q1)	Age, sex, smoking, exercise, SBP, DBP, waist ratio, ALT, AST, T. bili, T. cholesterol, DM	3.2 years
Khamseh, 2020 [12]	CS	Iran	General population	alcohol abuse, DM, viral hepatitis, chronic liver disease, pregnant or breastfeeding women	184	NR	44.7	30.5	96 (52.2)	NR	Ultrasound	Continuous	Age, gender, hip, SBP, DBP, ALT, AST, cholesterol, HOMA-IR, statin, smoking	NR
Kim, 2021 [23]	RC	Korea	General population	alcohol abuse, DM, viral hepatitis, HLD	10,585	6326 (59.8)	47.8	23.6	3284 (51.9)	NR	Ultrasound	Categorized (Q4:Q1)	NR	NR
Kitae, 2019 [24]	PC	Japan	General population	alcohol abuse, viral hepatitis, and chronic liver disease including NAFLD	14,086	6823 (48.4)	40	21.2	2670 (39.1)	NR	Ultrasound	Categorized (T3:T1)	Age, ALT, BMI, alcohol consumption, exercise, and smoking	1881 (IQR 2771) days in men and 2198 (IQR 2645) in women
Lee, 2019 [26]	CS	Korea	General population	alcohol abuse, hypertriglyceridemia, chronic liver disease, viral hepatitis, cancer, DM, acute inflammation, and renal or infectious disease	4986	2979 (59.7)	52.63	23.82	2069 (41.5)	NR	Ultrasound	Categorized (Q4:Q1)	Age, sex, BMI, SBP, TC, HDL, ALT, HTN	NR
Lin, 2021 [27]	CS	Taiwan	General population	alcohol abuse and viral hepatitis	1969	764 (38.4)	55.1	25.3	826 (42)	NR	Ultrasound	Continuous	Age, AST, ALT, T. Cholesterol, Hemoglobin, GFR, uric acid	NR
Malek, 2021 [15]	CS	Iran	Patients with T2DM	alcohol abuse, hepatitis, chronic liver disease, liver masses, and pregnant or breastfeeding women	175	80 (45.7)	48.3	29.55	122 (69.7)	NR	Elastography (FibroScan)	Categorized (Q4:Q1)	age, gender	NR
Riviere, 2022 [25]	Cohort	France	Patients with obesity	alcohol abuse, DM except T2DM, previous bariatric surgery except LAGB, chronic inflammatory disease and cancer	238	80 (33.6)	43	42	160 (67.2)	68 (28.6%)	Liver biopsy	Continuous	sex, age, BMI, AST, GGT	NR
Sheng, 2021 [31]	CS	China	General population	alcohol abuse, chronic liver disease including NAFLD, viral hepatitis, and impaired glucose tolerance	14,251	7411 (52)	44.6	23.45	2507 (17.6)	NR	Ultrasound	Continuous	GGT, age, drinking status, HbA1c, TC, smoking status, HDL-C, habit of exercise, and DBP	NR
Taheri, 2022 [11]	RC	Iran	General population	alcohol abuse, cancer, IBD, transplant patients, viral hepatitis, chronic liver disease, pregnant or breastfeeding women	1932	756 (39.1)	48.98	28.54	968 (50.1)	NR	fatty liver index	Categorized (T1–T3)	age, sex, smoking, physical activity level, WHR, SPB, DBP, ALT, and TC levels	NR
Wang, 2021 [28]	CS	China	General population (women)	alcohol abuse and viral or autoimmune hepatitis	3239	None	58.2	25.4	2257 (69.7)	NR	Ultrasound	Categorized (Q1–Q4)	Age, medical history, SBP, DBP, BMI, RBC, WBC, PLT, FPG, HbA1c, ALT, AST, ALP, BUN, GGT, TBIL, Cr, BUN, UA	NR
Xie, 2021 [29]	CS	China	General population	alcohol abuse and chronic liver disease	1748	1153 (66)	44.5	24.6	526 (30.1)	NR	Ultrasound	Categorized (Q1–Q4)	Age and gender	NR
Zhang, 2017 [10]	CS	China	General population	alcohol abuse, viral hepatitis, chronic liver disease, DM and HLD	10,761	6758 (62.8)	49.5	23.9	4349 (64.4)	NR	Ultrasound	Categorized (Q1–Q4)	Age, gender, ALT	NR
Zheng, 2018 [30]	PC	China	General population	alcohol abuse, chronic liver disease, HTN, DM, HLD, and multiple sclerosis	4539	2996 (66)	41	22.7	1390 (30.6)	NR	Ultrasound	Categorized (Q4:Q1)	BMI, WC, gender, SBP, age, DBP, TC, TG, Apo-A1, Apo-B, LDL-C, FPG, BUN, Cr, HDL-C, AST, ALT, UA, y-GGT and eGFR	9 years

Abbreviations: ALT: alanine transaminase, AST: aspartate transaminase, BUB: blood urea nitrogen, BMI: body mass index, CKD: chronic kidney disease, Cr: creatinine, CS: cross-sectional, DM: diabetes mellitus, DBP: diastolic blood pressure, ESRD: end-stage renal disease, HOMA-IR: homeostatic model assessment of insulin resistance, HLD: hyperlipidemia, HTN: hypertension, NAFLD: nonalcoholic fatty liver disease, NR: not reported, PLT: platelet count, PC: prospective cohort, RC: retrospective cohort, SBP: systolic blood pressure, TBIL: total bilirubin, TC: total cholesterol, TG: triglyceride, TyG index: triglyceride-glucose index, T2DM: type 2 diabetes mellitus, WC: waist circumference, and WHR: waist–hip ratio.

## Data Availability

The authors declare that all the data supporting the findings of this study are available within the manuscript.

## Supplementary Materials

The following supporting information can be downloaded at: https://www.mdpi.com/article/10.3390/jcm11092666/s1, Figure S1: Leave-one-out sensitivity analysis for the association between TyG index (analyzed as a categorical variable and presented as adjusted odds ratio) and incident NAFLD. Table S1: Search strategy used in each database searched. Table S2: Quality assessment of the included studies in the meta-analysis.
