# Supplementary material for: Triglyceride-Glucose Index for Early Prediction of Nonalcoholic Fatty Liver Disease: A Meta-Analysis of 121,975 Individuals
Source: J Clin Med. 2022 May 9;11(9):2666. doi: 10.3390/jcm11092666 (PMC9102411; doi:10.3390/jcm11092666)
Supplement: Supplementary file 1 [file jcm-11-02666-s001.zip › jcm-1701178-supplementary.pdf]

Supplementary Appendix

Supplementary tables

Title	Page
Supplementary Table S1: Search strategy used in each database searched.	2
Supplementary Table S2: Quality assessment of the included studies in the meta-analysis.	3-4

Supplementary figures:

Title	Page
Supplementary Figure S1: Leave-one-out sensitivity analysis for the association between TyG index (analyzed as a categorical variable and presented as adjusted odds ratio) and incident NAFLD.	5

Supplementary Table S1: Search strategy used in each database searched.

Database	Search Strategy	Articles retrieved
PubMed/MEDLINE	(("triglyceride-glucose"[All Fields] AND ("abstracting and indexing"[MeSH Terms] OR ("abstracting"[All Fields] AND "indexing"[All Fields]) OR "abstracting and indexing"[All Fields] OR "index"[All Fields] OR "indexed"[All Fields] OR "indexes"[All Fields] OR "indexing"[All Fields] OR "indexation"[All Fields] OR "indexations"[All Fields] OR "indexe"[All Fields] OR "indexer"[All Fields] OR "indexers"[All Fields] OR "indexs"[All Fields])) OR ("TYG"[All Fields] AND ("abstracting and indexing"[MeSH Terms] OR ("abstracting"[All Fields] AND "indexing"[All Fields]) OR "abstracting and indexing"[All Fields] OR "index"[All Fields] OR "indexed"[All Fields] OR "indexes"[All Fields] OR "indexing"[All Fields] OR "indexation"[All Fields] OR "indexations"[All Fields] OR "indexe"[All Fields] OR "indexer"[All Fields] OR "indexers"[All Fields] OR "indexs"[All Fields]))) AND ("non alcoholic fatty liver disease"[MeSH Terms] OR ("non alcoholic"[All Fields] AND "fatty"[All Fields] AND "liver"[All Fields] AND "disease"[All Fields]) OR "non alcoholic fatty liver disease"[All Fields] OR ("non"[All Fields] AND "alcoholic"[All Fields] AND "fatty"[All Fields] AND "liver"[All Fields] AND "disease"[All Fields]) OR "non alcoholic fatty liver disease"[All Fields] OR ("naflds"[All Fields] OR "non alcoholic fatty liver disease"[MeSH Terms] OR ("non alcoholic"[All Fields] AND "fatty"[All Fields] AND "liver"[All Fields] AND "disease"[All Fields]) OR "non alcoholic fatty liver disease"[All Fields] OR "nafld"[All Fields]) OR "NASH"[All Fields])	57
Embase	('triglyceride-glucose index' OR ('triglyceride glucose' AND ('index'/exp OR index)) OR 'tyg index' OR (tyg AND ('index'/exp OR index))) AND ('non-alcoholic fatty liver disease'/exp OR 'non-alcoholic fatty liver disease' OR ('non alcoholic' AND fatty AND ('liver'/exp OR liver) AND ('disease'/exp OR disease)) OR nafld OR nash)	102
Web of Science	(triglyceride-glucose index OR TYG index) AND (non-alcoholic fatty liver disease OR NAFLD OR NASH)	62

Supplementary Table S2: Quality assessment of the included studies in the meta-analysis.

Observational studies	Selection				Comparability	Outcome			Quality score
	Representativeness of the exposed cohort	Selection of non-exposed cohort	Ascertainment of exposure	Demonstration that outcome of interest was not present at start of study	Comparability of the cohorts on the basis of design or analysis	Assessment of outcome	Was follow up long enough for outcomes to occur	Adequacy of follow up cohorts	
Celik, 2021	1	1	1	1	1	1	0	1	7
Choe, 2020	0	1	1	1	1	1	0	1	6
Guo, 2020	1	1	1	1	1	1	0	1	7
Huanan, 2020	1	1	1	1	1	1	1	1	8
Khamseh, 2020	1	1	1	1	1	1	0	1	7
Kim, 2021	1	1	1	1	1	1	0	1	7
Kitae, 2019	1	1	1	1	1	1	1	1	8
Lee, 2019	1	1	1	1	1	1	0	1	7
Lin, 2021	1	1	1	1	1	1	0	1	7
Malek, 2021	0	1	1	1	1	1	0	1	6
Riviere, 2022	0	1	1	1	1	1	0	1	6
Sheng, 2021	1	1	1	1	1	1	0	1	7
Taheri, 2022	1	1	1	1	1	1	0	1	7
Wang, 2021	1	1	1	1	0	1	0	1	6
Xie, 2021	1	1	1	1	1	1	0	1	7

Zhang, 2017	1	1	1	1	1	1	0	1	7
Zheng, 2018	1	1	1	1	1	1	1	1	8

Study name	Statistics with study removed				
	Point	Lower limit	Upper limit	Z-Value	p-Value
Celik, 2021	6.353	4.255	9.485	9.040	0.000
Guo, 2020	6.449	4.345	9.572	9.250	0.000
Kim, 2021	5.798	3.624	9.275	7.330	0.000
Lee, 2019	6.806	4.859	9.533	11.154	0.000
Malek, 2021	6.155	4.194	9.033	9.283	0.000
Taheri, 2022	5.436	3.706	7.972	8.664	0.000
Wang, 2021	6.181	4.070	9.388	8.542	0.000
Xie, 2021	5.159	3.628	7.337	9.134	0.000
Zhang, 2017	5.888	3.636	9.534	7.210	0.000
	6.004	4.121	8.746	9.337	0.000

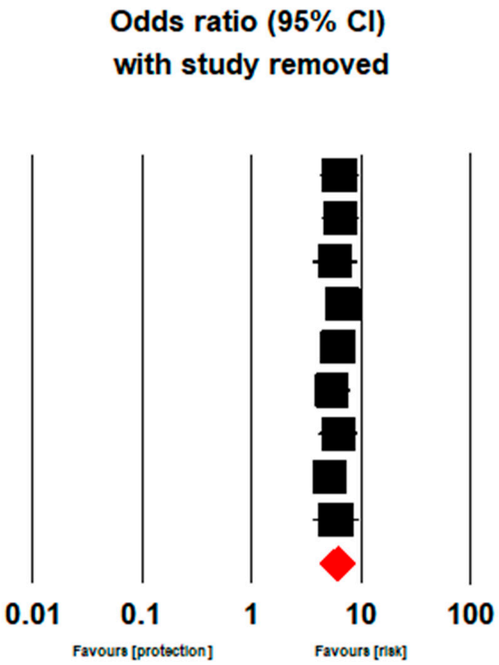

Supplementary Figure S1: Leave-one-out sensitivity analysis for the association between TyG index (analyzed as a categorical variable and presented as adjusted odds ratio) and incident NAFLD.
